# Exploring a Mental Fatigue Signal Hidden in GPS Data: Acute Pre-to-Post-Match Psychomotor Performance and Exploratory Associations with External Load in Professional Soccer

**DOI:** 10.3390/sports14070261

**Published:** 2026-06-24

**Authors:** Andreas Stafylidis, Walter Staiano, Athanasios Mandroukas, Yiannis Michailidis, Mert Isbilir, Lazaros Vardakis, Andreas Fousekis, Konstantinos Chatzinikolaou, Lluis Raimon Salazar Bonet, Ana Ferri-Caruana, Nikolaos Tsigilis, Marco Romagnoli, Thomas I. Metaxas

**Affiliations:** 1 Laboratory of Evaluation of Human Biological Performance, Department of Physical Education and Sports Sciences, University Campus of Thermi, Aristotle University of-Thessaloniki, 57001 Thessaloniki, Greece; amandrou@phed.auth.gr (A.M.); ioannimd@phed.auth.gr (Y.M.); vardakis@live.com (L.V.); antreasfousekis@gmail.com (A.F.); tommet@phed.auth.gr (T.I.M.); 2 Department of Physical Education and Sport, University of Valencia, 46000 Valencia, Spain; walterstaiano@gmail.com (W.S.); ana.maria.ferri@uv.es (A.F.-C.); marco.romagnoli@uv.es (M.R.); 3 Department of Physical Education and Sports Science, Democritus University of Thrace, 69100 Komotini, Greece; mert_isbilir@hotmail.com; 4 Laboratory of Motor Behavior and Adapted Physical Activity, Department of Physical Education and Sport Science, Aristotle University of Thessaloniki, 57001 Thessaloniki, Greece; konchatzinikolaou@phed.auth.gr; 5 International University SEK, Quito 170151, Ecuador; raimon.salazar@uisek.edu.ec; 6 School of Journalism and Mass Communications, Faculty of Economics and Political Sciences, Aristotle University of Thessaloniki, 54625 Thessaloniki, Greece; ntsigilis@jour.auth.gr

**Keywords:** mental fatigue, wearable technologies in sport, football, external load, psychomotor performance, reaction time, cognitive readiness, neurocognitive monitoring, psychophysiological responses, internal load

## Abstract

This study examined acute pre- to post-match changes in perceived mental fatigue, subjective workload, and psychomotor performance in professional male soccer players, and whether cognitive changes were associated with GPS-derived external-load metrics, match outcome, and playing position. The dataset comprised 101 player–match measurements from 40 elite players, with paired pre–post psychomotor assessments yielding *n* = 202 total measurements. Pre–post comparisons were analysed using repeated-measures ANOVA, supplemented by linear mixed-effects models with a random intercept for player. Soccer matches produced large increases in perceived exertion, mental fatigue, mental demand, physical demand, and effort (all *p* < 0.001), and significant deteriorations in reaction time, accuracy, processing speed, and response variability (all *p* ≤ 0.005), confirmed in the mixed-effects analyses (all *p* ≤ 0.014). In the initial player–match-level analyses, high-intensity accelerations (>3 m·s^−2^) were weakly associated with greater Δreaction-time slowing (r = 0.203), increased response variability (r = 0.276), and reduced Rate Correct Score (r = −0.242), while high metabolic load distance was weakly associated with post-match perceived mental fatigue but not with psychomotor-performance changes. One-way ANOVAs indicated greater post-match psychomotor decrements following losses than draws. Once within-player dependence was modelled, the effects of match outcome, playing position, and most external-load metrics were attenuated, except for a residual match-outcome effect on accuracy and a high-intensity deceleration effect on accuracy. These findings indicate that competitive soccer match play is followed by acute psychomotor-performance decrements and increased perceived mental fatigue, whereas the contributions of mechanical load, match outcome, and playing position appear modest and partly reflect stable between-player differences.

## 1. Introduction

Mental fatigue (MF) is commonly defined as a psychobiological state arising from sustained cognitively demanding activity and characterised by subjective sensations of tiredness or low energy, reduced motivation, and impairments in executive control and attention [1]. Conceptual models emphasise that MF reflects an altered cost–benefit evaluation of continued task engagement, leading to diminished effort investment and difficulties in maintaining focus and sustaining goal-directed behaviour [1]. A systematic review by Van Cutsem et al. [2] showed that experimentally induced MF—typically via prolonged response-inhibition or vigilance tasks of at least 30 min duration—reliably impairs subsequent physical performance, particularly endurance exercise, even when traditional cardiorespiratory markers are not substantially altered. Complementary work in adolescent endurance athletes confirms that prior mental exertion impairs both aerobic performance and cognitive outcomes on attention and executive-function tasks, while increasing perceived exertion during exercise [3].

Beyond global endurance, MF has important consequences for sport-specific psychomotor performance, understood as complex actions that integrate perception, decision-making and motor execution in dynamic environments [4]. The recent systematic scoping review in team sports concluded that MF consistently impairs sport-specific motor performance, with decrements observed in technical accuracy, tactical behaviour and decision-making across a range of experimental paradigms [4]. In soccer, where players must continuously process rapidly changing information about teammates, opponents, ball trajectory and space, these effects are particularly concerning [5]. Experimental studies have shown that MF induced by a 30 min Stroop task reduces the accuracy and increases the response time of film-based, soccer-specific decision-making tasks in well-trained players, indicating that a 30 min period of elevated cognitive demand can degrade the speed and quality of tactical choices [6]. Experimental evidence also suggests that MF extends beyond decision-making accuracy, as a 30 min Stroop task impaired directional repeated-sprint ability and repeated-jump performance in trained male team-sport athletes, including soccer players, alongside increased perceived exertion and reduced psychomotor vigilance, despite no corresponding changes in heart rate or blood lactate responses [7]. Small-sided game studies further demonstrate that prior MF can impair acceleration distance, increase technical errors such as unsuccessful passes, and reduce passing and shooting accuracy in soccer-specific contexts, although GPS-derived total distance and high-speed running distance showed unclear changes between conditions [8].

These findings intersect with a broader literature on perceptual-cognitive expertise in soccer. Skilled players typically outperform less-skilled counterparts in anticipation and decision-making tasks, underpinned by more efficient visual search strategies (more fixations of shorter duration to informative locations), better use of situational probabilities, and superior integration of postural cues and pattern recognition [9,10,11]. Creative and effective decision makers also exhibit a broader and more flexible attentional focus, employing more fixations of shorter duration directed towards more informative locations of the display, enabling them to detect teammates in threatening positions earlier in attacking sequences [10]. Because these abilities depend heavily on sustained attention, working memory, and inhibitory control, theoretical accounts and emerging empirical data may suggest that MF may undermine anticipation, focus and decision-making in soccer by selectively degrading the very executive functions and vigilance processes that support high-level perceptual-cognitive performance [1,2,6].

Professional soccer places substantial and evolving demands on players’ physical and cognitive systems, with elite match play characterised by high volumes of intermittent high-intensity activity, extensive high-speed running and repeated sprint efforts superimposed on a large aerobic running base [12,13]. Longitudinal tracking data from the English Premier League show that, over seven seasons, high-intensity running distance increased by approximately 30% (from 890 ± 299 m to 1151 ± 337 m) and sprint distance increased by approximately 35% (from 232 ± 114 m to 350 ± 139 m), despite only minimal changes in total distance covered, illustrating a shift towards more intense locomotor profiles at the professional level [12]. Within matches, these external-load demands are modulated by playing position and tactical role, with wide defenders and wide midfielders typically covering the greatest sprint and high-speed running volumes, while central players perform more sustained running and technical actions [13,14]. Match-analysis work has also highlighted that players undertake frequent high-intensity accelerations and decelerations, which contribute substantially to mechanical and metabolic stress despite often occurring at submaximal running speeds [15,16]. To quantify and manage these complex movement demands, elite clubs have widely adopted global positioning system (GPS) technology to monitor external load in training and matches, with current practice surveys indicating near-universal use in high-level football/soccer environments [17,18].

External-load monitoring in professional soccer typically focuses on total distance, high-speed running (HSR), sprint distance, and the frequency or magnitude of accelerations and decelerations as key metrics summarising the mechanical and energetic cost of sessions [17,19]. HSR and sprint distance are of particular interest because they are strongly linked to decisive match incidents and have increased substantially in modern play [12,19]. Although HSR and sprinting thresholds vary considerably across adult soccer studies, with increasing support for individualised cut-offs based on maximal sprinting speed, these GPS-derived external-load metrics are generally supported by validation evidence showing acceptable reliability and validity for measuring velocity, distance, accelerations, and decelerations during match-like movement demands [20,21,22,23,24].

Fatigue in football is inherently multifactorial and arises from the interaction of physiological, biomechanical and psychological stressors over congested competition schedules and high-load training cycles [25,26]. Traditionally, most applied and research attention has focused on physical or physiological fatigue, conceptualised as the reduction in the ability to produce force or power due to peripheral and/or central mechanisms, and operationalised through declines in sprint speed, jumps, or high-intensity running outputs during or after matches [25]. However, contemporary frameworks increasingly emphasise that these physical expressions of fatigue occur in parallel with, and are influenced by, mental or cognitive fatigue states that can modulate perception of effort, motivation and decision-making during high-level competition [2,4,6,25,27]. Experimental work using demanding response-inhibition or vigilance tasks has demonstrated that MF does not substantially alter traditional cardiorespiratory or peripheral neuromuscular markers during subsequent exercise, but instead increases ratings of perceived exertion at a given work rate and hastens the attainment of maximal perceived effort [27]. Within the psychobiological model of endurance performance, MF is proposed to raise the perceived effort associated with motor commands without necessarily changing afferent feedback, thereby reducing exercise tolerance even when physiological capacity is preserved [2,27].

In team-sport contexts, accumulating evidence indicates that MF also degrades sport-specific cognitive and technical performance, with soccer representing one of the most studied models [2,4,25]. In intermittent running tests designed to replicate team-sport demands, mentally fatigued players exhibit reduced running velocities at lower exercise intensities and higher perceived exertion compared with control conditions, despite similar heart rate and lactate responses [28]. When MF is induced before soccer-specific simulations or small-sided games, players demonstrate impaired acceleration distance, greater technical error rates, and diminished passing and shooting accuracy, highlighting concomitant physical and skilled decrements [6,8]. Game-based research further indicates that MF modifies tactical behaviour and spatial coordination, with mentally fatigued players demonstrating reduced synchronisation with teammates in the lateral direction and altered speed of team contraction, even when total running distance showed only unclear between-condition differences [29]. Together, these data suggest that MF can influence not only players’ capacity to perform high-intensity actions, but also how they perceive and interact with the dynamic informational constraints of the game.

Psychomotor vigilance and reaction-time tasks are widely regarded as sensitive markers of MF and sustained attention, and they provide simple, objective indices that can be deployed repeatedly in applied environments [30,31]. The conventional psychomotor vigilance test (PVT) is a simple reaction-time paradigm in which participants respond as quickly as possible to visual stimuli presented at random inter-stimulus intervals, with key performance metrics including mean reaction time, the number of lapses (very slow responses), and variability in response times [30,31,32]. Sleep-deprivation research has demonstrated that MF and reduced alertness are reliably associated with overall slowing of responses, increased lapses, and greater time-on-task deterioration on the PVT, establishing these metrics as well-established indicators of cognitive fatigue and vigilant attention deficits [30,31]. To improve practicality in field contexts, brief PVT variants (PVT-B) of approximately three minutes have been developed and validated, showing acceptable sensitivity to sleep loss and MF while substantially reducing testing time [33].

App-based platforms can implement PVT-like paradigms on mobile devices, enabling the assessment of psychomotor vigilance and related cognitive constructs alongside physical load monitoring in applied sport settings [30,31]. Experimental work combining mentally fatiguing cognitive tasks with subsequent physical and cognitive testing has shown that brief PVT protocols are sensitive to MF, with MF conditions eliciting slower reaction-times and more lapses compared with control conditions, alongside impaired intermittent running and sprint performance [34,35,36]. In these studies, mean reaction time, the number of lapses (responses above a predefined latency threshold), and response time variability have been used as primary behavioural markers of MF, consistent with recommendations from the broader PVT and fatigue literature [30,31,34]. In addition to PVT-B-style protocols, some app-based systems also include a 90 s Psychomotor Fatigue Threshold Test (PFTT), implemented as a choice reaction time task, which has been used as a behavioural measure of brief psychomotor performance in exercise settings [37]. Collectively, these mobile implementations of psychomotor vigilance and psychomotor fatigue tests offer a practical means of repeatedly assessing MF and cognitive performance in sport environments where testing time and logistical constraints are substantial.

Although several experimental studies have examined how acutely induced MF influences soccer-specific running, technical execution, decision-making, and tactical behaviour, this evidence has largely been derived from controlled fatigue manipulations, such as prolonged Stroop or response-inhibition tasks, followed by laboratory-based, standardised, or small-sided-game assessments [4,6,8,28,29]. These studies have provided important mechanistic and sport-specific evidence, but they do not fully explain how psychomotor performance fluctuates in response to the routine external loads imposed by competitive match play in professional players.

This gap is relevant because contemporary training-load frameworks emphasise the need to understand how athletes’ internal states respond to external demands in order to optimise adaptation, manage fatigue, and reduce injury risk [18,26,38,39,40]. However, longitudinal and ecologically valid evidence remains limited regarding the association between objective cognitive-performance measures—such as reaction time, response variability, accuracy, and integrated speed–accuracy indices—and GPS-derived external-load profiles in professional male soccer, particularly when mental fatigue and psychomotor performance are assessed both before and immediately after matches.

Accordingly, the present study examined acute pre- to post-match changes in perceived mental fatigue, subjective workload, and brief psychomotor performance in professional male soccer players. The primary confirmatory aim was to determine whether competitive match play was followed by increased perceived exertion, perceived mental fatigue, and subjective workload, together with deterioration in psychomotor performance, expressed as slower reaction time, reduced accuracy, lower processing speed, greater response-time variability, and reduced Rate Correct Score. Based on existing psychobiological and soccer-specific literature [1,2,4,6], it was hypothesised that these post-match changes would be observed.

As secondary exploratory analyses, the study examined whether the magnitude of psychomotor change was associated with GPS-derived external-load metrics recorded during competitive matches, including locomotor volume, high-speed and sprint running, high metabolic load distance, maximal speed, and high-intensity acceleration and deceleration actions. In addition, exploratory analyses examined whether psychomotor changes differed according to match outcome and playing position, and whether selected match-load and contextual variables explained the magnitude of post-match psychomotor deterioration.

## 2. Materials and Methods

### 2.1. Participants

The study included 40 professional male soccer players who participated in competitive matches and completed cognitive and perceptual assessments before and after competition. The final dataset comprised 101 player–match measurements collected across 12 competitive matches from 40 elite male players, each providing paired pre- and post-match cognitive assessments (*n* = 202). Players were 22.73 ± 4.35 years old, with a training age of 15.18 ± 2.72 years. Mean height was 1.79 ± 0.06 m, body mass was 75.00 ± 6.46 kg, and body mass index was 23.31 ± 1.37 kg·m^−2^. Mean body fat was 8.89 ± 1.58%, fat-free mass was 68.20 ± 5.47 kg, fat mass was 6.72 ± 1.56 kg, and resting heart rate was 60.60 ± 6.89 bpm. Twenty-five players were right-footed (62.5%) and 15 were left-footed (37.5%).

Players were classified according to their usual playing position as central defenders (*n* = 10, 25.0%), central midfielders (*n* = 8, 20.0%), forwards (*n* = 6, 15.0%), side defenders (*n* = 10, 25.0%), and side midfielders (*n* = 6, 15.0%). Goalkeepers were excluded from the final dataset. The team competed in the national championship. In accordance with the participant classification framework proposed by McKay et al. [41], the athletes were classified between Tier 3 and Tier 4, corresponding to highly trained/national-level to elite/international-level athletes. This classification was based on the competitive level of the team, the players’ professional status, and their participation in national-level competition. All players were free from injury and illness at the time of testing and were eligible for match participation. Before data collection, participants were informed about the purpose and procedures of the study and provided written informed consent. Ethical approval was obtained from the Ethics Committee of the School of Physical Education and Sport Science at Aristotle University of Thessaloniki (Approval No. 192/2024), and all participants provided written informed consent.

### 2.2. Anthropometric and Resting Cardiovascular Measurements

Anthropometric and body-composition variables were assessed in the morning using standardised procedures. Body mass, body fat percentage, fat-free mass, fat mass, and muscle mass were measured with a TANITA DC-360 analyser (Tanita Corporation, Tokyo, Japan), based on bioelectrical impedance analysis. Before testing, players were instructed to maintain normal hydration and avoid food and caffeine intake. Body height was recorded to the nearest 0.1 cm using a Seca 220e stadiometer (Seca, Hamburg, Germany). All measurements were performed with participants barefoot and wearing minimal clothing. Resting cardiovascular measures were obtained under standardised conditions. Participants were placed in a quiet, isolated room and remained lying supine for 5 min before measurement. Resting heart rate was subsequently recorded using an automated sphygmomanometer (M7, Omron, Vernon Hills, IL, USA). The cuff was positioned at heart level, with the measurement site aligned approximately with the right atrium. Two readings were taken, and the average value was retained for analysis.

### 2.3. Study Design

A repeated-measures observational design was used to examine changes in psychomotor performance, perceived mental fatigue, motivation, and subjective workload from pre-match to post-match. In addition, the study investigated whether the magnitude of pre- to post-match changes in cognitive performance was associated with external match-load variables, playing position, and match outcome.

**Figure 1:**
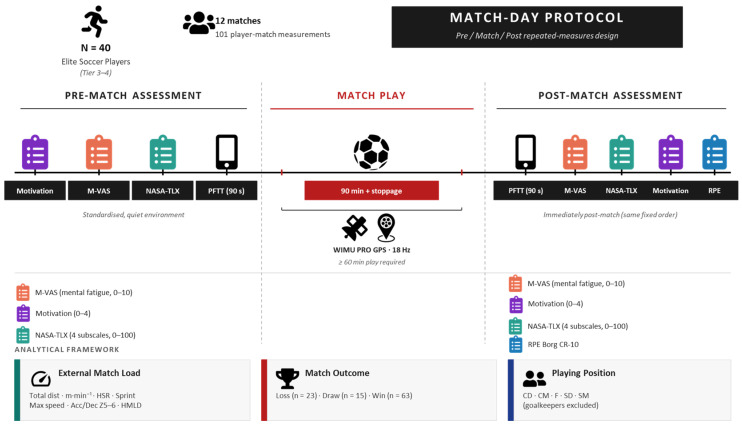
Match-day assessment protocol and analytical framework of the study. Note. Overview of the repeated-measures match-day protocol implemented in 40 elite male soccer players across 12 matches, yielding 101 player–match measurements. Before match play, players completed assessments of motivation, mental fatigue (M-VAS), subjective workload (NASA-TLX), and a 90 s Psychomotor Fatigue Threshold Test (PFTT) in a standardised quiet environment. During match play (90 min plus referee-added stoppage time), external load was monitored using a WIMU PRO GPS device (18 Hz). Only measurements with valid match-load recordings and players who completed at least 60 min of match play were retained for the external-load analyses. Immediately after the match, players repeated the assessment battery in the following fixed order: PFTT, M-VAS, NASA-TLX, motivation, and RPE. The analytical framework included associations between pre- to post-match cognitive changes and external match load, as well as comparisons according to match outcome and playing position.

Each player completed the same assessment battery before and after the match (Figure 1). The pre-match assessment was conducted under standardised conditions before the beginning of match-related activity, whereas the post-match assessment was completed within 5 min after the final whistle to capture the acute psychophysiological responses associated with competitive match play. Match outcome was classified as loss, draw, or win. External load was monitored during match play using a wearable tracking system, and the extracted variables were subsequently used in the correlational and regression analyses.

### 2.4. Procedures

Before the match, players completed subjective ratings of motivation and mental fatigue and then performed the psychomotor vigilance assessment using the SOMA-NPT application (Soma Technologies, Lucerne, Switzerland), which has been used in several peer-reviewed studies [7,35,36,42,43,44]. The pre-match testing session was conducted in a quiet environment to minimise external distractions and to ensure consistency across players.

Following the match, players repeated the same perceptual and cognitive assessments. The post-match assessment battery was administered in the following order: the 90 s Psychomotor Fatigue Threshold Test (PFTT), Mental Visual Analog Scale, selected NASA Task Load Index subscales, motivation scale and rating of perceived exertion. The order of administration was kept identical between the pre- and post-match assessments to minimise procedural variability.

During match play, players wore the same tracking device positioned according to the manufacturer’s instructions. Match-running performance was quantified using total distance, distance covered per minute, high-speed running distance, sprint distance, combined high-speed running plus sprint distance, maximal speed, high-intensity accelerations and decelerations, and high metabolic load distance.

### 2.5. Cognitive and Perceptual Measures

Objective psychomotor performance, used as a behavioural indicator of mental fatigue, was assessed using the 90 s Psychomotor Fatigue Threshold Test (PFTT), administered via the SOMA-NPT application installed on an iOS smartphone. The PFTT is a computerised choice reaction time task in which participants are exposed to 25 visual stimuli over a 90 s period, consisting of 10 green circles, 10 red circles, and 5 yellow circles. Participants were instructed to respond as quickly and accurately as possible by tapping the left on-screen button when a green circle appeared and the right on-screen button when a red circle appeared, while withholding any response when a yellow circle was presented. The PFTT has previously been used in soccer and endurance-sport contexts as an applied index of psychomotor readiness and fatigue-related response slowing [45,46]. Given its short duration and simple stimulus–response structure, the PFTT was treated as a brief behavioural marker of psychomotor performance rather than as a comprehensive assessment of higher-order cognition. Therefore, the task does not reproduce the sport-specific perceptual-cognitive demands of match play and should not be interpreted as a direct measure of soccer decision-making or executive function. Mean reaction time for correct responses, expressed in milliseconds and calculated automatically by the SOMA-NPT software, was used as the primary behavioural outcome. Higher reaction-time values were interpreted as slower psychomotor responding, particularly when considered together with the remaining cognitive and perceptual outcomes. Moreover, this SOMA-NPT PFTT protocol has recently been applied in exercise-related studies as a standardised measure of psychomotor performance and fatigue-related cognitive function [37,47]. The SOMA-NPT output provided reaction time, accuracy, response variation, speed, and Rate Correct Score. Reaction time reflected the average latency of correct responses, whereas accuracy represented the percentage of correct responses. Response variation described intra-individual inconsistency in reaction time across trials. Speed was automatically calculated as 1000 divided by reaction time, thereby providing an inverse index of response speed. Rate Correct Score represented the number of correct responses per second and was used as an integrated speed–accuracy indicator of overall psychomotor efficiency.

Subjective mental fatigue was assessed using the Mental Visual Analog Scale (M–VAS). Players indicated their perceived level of mental fatigue on a 10 cm horizontal line anchored by “not at all exhausted” and “completely exhausted.” The same scale was administered before and after match play, following procedures previously described in the literature [7,35,36,48].

Subjective workload was quantified using four dimensions derived from the NASA Task Load Index (NASA-TLX): mental demand, physical demand, effort, and frustration [49]. Consistent with previous sport and exercise studies using selected NASA-TLX dimensions [7,35,36], players provided separate ratings for each dimension immediately before and after match play. Each item was scored on a 20-step response scale and subsequently converted to a 0–100 score by multiplying the raw rating by five. Thus, higher values indicated greater perceived workload for the corresponding dimension. The four subscales were analysed separately rather than combined into a global NASA-TLX score.

Motivation to perform the assessment was measured using a 5-point Likert scale. Players responded to the statement, “I am motivated to perform the test,” using a scale ranging from 0, indicating “not at all,” to 4, indicating “extremely.” This approach is consistent with previous protocols used to quantify motivation during physical and cognitive performance testing [34,35,36].

Rating of perceived exertion (RPE) was assessed using the Borg CR-10 scale [50,51], as used in previous similar studies [34,35,36]. Players rated their perceived effort on a scale from 0 to 10, where 0 indicated no exertion at all and 10 indicated maximal exertion.

### 2.6. External Match-Load Measures

External match load was recorded during match play using the WIMU PRO Global Positioning System device operating at 18 Hz (Realtrack Systems S.L., Almería, Spain). The validity and reliability of WIMU PRO devices and related GPS-based tracking systems for quantifying movement demands in team-sport settings have been reported previously [52,53], and the same WIMU PRO system has also been applied in recent soccer match-analysis research [54]. Only measurements from players who completed at least 60 min of match play were retained for analyses of both absolute external-load variables and relative indicators, particularly distance·min^−1^. Match duration consisted of 90 min plus any additional stoppage time determined by the referee.

External-load variables were derived from the WIMU GPS and included high-speed running distance, sprint distance, high-speed running plus sprint distance, and high-intensity acceleration and deceleration counts. High-speed running distance (HSR, m) was calculated as the distance covered at speeds exceeding 19.8 km·h^−1^, while sprint distance (SD, m) was calculated as the distance covered at speeds exceeding 25.2 km·h^−1^. The composite variable HSR + SD (m) was calculated as the sum of HSR and SD. High-intensity accelerations and decelerations were quantified as the number of actions exceeding 3 m·s^−2^ and −3 m·s^−2^, respectively.

### 2.7. Statistical Analysis

A priori power analyses were conducted using G*Power 3.1.9.7 [55,56] for the planned analyses. For the pre–post comparisons, the “Means: Difference between two dependent means (matched pairs)” procedure was used. Assuming a two-tailed α = 0.05, 80% power, and a moderate effect size of dz = 0.40, the analysis indicated that 52 complete paired measurements were required. For bivariate correlations, the “Correlation: Bivariate normal model” procedure indicated that 84 measurements were required to detect a moderate association of r = 0.30 with α = 0.05 and 80% power. The final dataset comprised 101 complete pre–post player–match measurements, exceeding these requirements for detecting moderate time effects and bivariate associations.

Data are presented as mean (standard deviation) unless otherwise stated. Normality was assessed using the Shapiro–Wilk test, together with visual inspection of histograms, Q–Q plots, and boxplots. Pre–post-match differences were examined using repeated-measures analyses of variance (RM-ANOVA), with time (pre-match, post-match) as the within-subjects factor. The main dependent variables included reaction time (ms), accuracy (%), speed, response variation (%), RCS (Rate Correct Score), motivation, M-VAS, and NASA-TLX subscales (mental demand, physical demand, effort, frustration).

Percentage change scores (Δ) were calculated as [(post-match − pre-match)/pre-match] × 100 to quantify the relative pre-to-post-match change in cognitive performance. The percentage change variables included ΔReaction time, ΔSpeed (percentage change in processing speed score from the cognitive battery), ΔVariation, ΔRCS, and ΔAccuracy. Positive Δ values indicate an increase and negative values a decrease relative to the pre-match measurement. One-way ANOVAs examined whether these change scores differed according to match outcome (win, draw, loss) and playing position. When significant main effects were observed, Bonferroni-adjusted post hoc comparisons were performed.

Pearson’s correlation coefficients were calculated to examine associations among changes in cognitive performance, perceptual responses, and external match-load variables. Correlations were interpreted according to Cohen’s [57] criteria, with magnitudes of 0.10, 0.30, and 0.50 considered small, medium, and large, respectively. Multiple linear regression models were conducted to examine whether selected match-load variables, playing position, and match outcome explained variance in cognitive change scores. For the ΔReaction time model, predictors included distance per minute, high-speed running plus sprint distance, high-intensity accelerations (>3 m·s^−2^), high-intensity decelerations (<−3 m·s^−2^), playing position, and match outcome. For the ΔVariation model, predictors were limited to GPS load metrics (distance per minute, high-speed running plus sprint distance, high-intensity accelerations (>3 m·s^−2^), high-intensity decelerations (<−3 m·s^−2^)).

To address the non-independence of repeated player–match measurements nested within players, linear mixed-effects models (LMM) were conducted as sensitivity analyses for the primary psychomotor outcomes using the GAMLj module in Jamovi [58,59]. Each model included time (pre-match, post-match) as a fixed effect, with high-intensity accelerations (>3 m·s^−2^), high-intensity decelerations (<−3 m·s^−2^), distance per minute, and high-speed running plus sprint distance entered as additional fixed predictors, alongside playing position and match outcome. A random intercept for player identity was included in all models to account for between-player variance [60]. Models were fitted by restricted maximum likelihood (REML) with the bobyqa optimizer; all models converged, and Wald confidence intervals are reported for fixed effects. Degrees of freedom were estimated using the Satterthwaite approximation. Model fit was evaluated using marginal *R*^2^ (variance explained by fixed effects) and conditional *R*^2^ (variance explained by fixed and random effects combined) [61], and the intraclass correlation coefficient (ICC) was reported to quantify the proportion of total variance attributable to between-player differences. Random intercepts for match were not modelled given the limited number of matches (k = 12), which is insufficient for reliable variance estimation at that clustering level.

All statistical analyses were performed using Jamovi (Version 2.6) [58], SPSS (Version 29.0.2; IBM Corporation, Armonk, NY, USA) [62], and JASP (Version 0.19.3) [63]. Effect sizes for RM-ANOVA and one-way ANOVA models were expressed as partial eta squared (η^2^p) and interpreted as small (≥0.01), medium (≥0.06), or large (≥0.14) according to Richardson [64]. Cohen’s *d* was used for pairwise comparisons and interpreted as small (0.20), medium (0.50), or large (0.80) [57], and, for pre–post comparisons, was calculated as the standardised mean difference using the pooled standard deviation of the two repeated-measures conditions. Effect size confidence intervals for η^2^p were calculated at the 90% level, consistent with recommendations for one-sided F-tests [65,66]; 95% confidence intervals were reported for Cohen’s *d* [65,67,68]. Confidence intervals for Pearson’s correlations were reported as 95% intervals throughout. Statistical significance was set at *p* < 0.05.

## 3. Results

### 3.1. Pre- to Post-Match Changes in Perceived Exertion, Mental Fatigue and Workload

Perceived exertion and mental fatigue were assessed using the rating of perceived exertion scale (RPE; 0–10) and the mental fatigue visual analogue scale (M-VAS; 0–10), respectively (Table 1 and Table 2). RPE increased markedly from pre- to post-match (*M*pre = 1.47, *SD* = 0.61; *M*post = 8.95, *SD* = 0.79), *F*(1, 100) = 5378, *p* < 0.001, η^2^p = 0.982, *d* = −10.59 [95% CI: −12.11, −9.08]. M-VAS scores increased from *M* = 3.52 (*SD* = 0.91) to *M* = 8.48 (*SD* = 0.96), *F*(1, 100) = 1777, *p* < 0.001, η^2^p = 0.947, *d* = −5.31 [95% CI: −6.10, −4.53], indicating that players reported substantially greater perceived exertion and mental fatigue after competitive matches.

All four NASA-TLX subscales were significantly elevated post-match (Table 2). Mental demand increased from *M* = 38.1 (*SD* = 9.0) to *M* = 83.0 (*SD* = 10.5), *F*(1, 100) = 1187, *p* < 0.001, η^2^p = 0.922, *d* = −4.57 [95% CI: −5.27, −3.88]; physical demand from *M* = 31.3 (9.5) to *M* = 88.7 (8.6), *F*(1, 100) = 2117, *p* < 0.001, η^2^p = 0.955, *d* = −6.37 [95% CI: −7.30, −5.43]; effort from *M* = 30.8 (10.3) to *M* = 85.8 (9.9), *F*(1, 100) = 1584, *p* < 0.001, η^2^p = 0.941, *d* = −5.45 [95% CI: −6.26, −4.64]; and frustration from *M* = 25.1 (*SD* = 8.4) to *M* = 29.5 (*SD* = 11.7), *F*(1, 100) = 18.75, *p* < 0.001, η^2^p = 0.158, *d* = −0.44 [95% CI: −0.65, −0.23]. Motivation did not change significantly (*M*pre = 3.76 vs. *M*post = 3.67; *F*(1, 100) = 2.66, *p* = 0.106, η^2^p = 0.026).

### 3.2. Pre- to Post-Match Changes in Cognitive Performance

Significant pre- to post-match decrements were observed across all cognitive performance metrics (Table 1). Reaction time slowed from *M* = 389.7 ms (*SD* = 61.5) to *M* = 438.9 ms (*SD* = 94.1), a mean increase of 49.1 ms, 95% CI [34.4, 63.8], and *F*(1, 100) = 43.82, *p* < 0.001, η^2^p = 0.305, *d* = −0.62 [95% CI: −0.82, −0.41], a moderate-to-large effect. Accuracy declined from *M* = 93.7% (*SD* = 7.3) to *M* = 91.1% (*SD* = 9.3), and *F*(1, 100) = 8.41, *p* = 0.005, η^2^p = 0.078, *d* = 0.32 [95% CI: 0.10, 0.54], a small but statistically significant decline. Processing speed decreased from *M* = 2.73 (*SD* = 0.41) to *M* = 2.51 (*SD* = 0.39), and *F*(1, 100) = 39.87, *p* < 0.001, η^2^p = 0.285, *d* = 0.53 [95% CI: 0.35, 0.71]. Response consistency worsened substantially, with variation increasing from *M* = 19.8% (*SD* = 7.6) to *M* = 26.5% (*SD* = 10.7), and *F*(1, 100) = 27.82, *p* < 0.001, η^2^p = 0.218, *d* = −0.72 [95% CI: −1.01, −0.43], indicating greater response-to-response inconsistency post-match.

### 3.3. Associations Between GPS-Derived External Load and Cognitive Performance Changes

Pearson correlations between GPS external load metrics and cognitive change scores are displayed in Table 3. Volume metrics—total distance, distance·min^−1^, HSR and sprint distances, and high metabolic load distance—were not significantly associated with any cognitive change variable (all rs ≤ 0.175, all *p*s > 0.09). High-intensity accelerations (>3 m·s^−2^) were significantly associated with ΔReaction Time% (r = 0.203, *p* = 0.042, 95% CI [0.007, 0.383]), ΔRCS% (r = −0.242, *p* = 0.015, 95% CI [−0.417, −0.049]), and ΔVariation% (r = 0.276, *p* = 0.005, 95% CI [0.085, 0.447]), indicating that greater acceleration load was associated with greater reaction-time slowing, lower speed–accuracy efficiency, and increased response variability. High-intensity decelerations (<−3 m·s^−2^) were significantly associated with ΔRCS% only (r = −0.238, *p* = 0.017, 95% CI [−0.414, −0.045]). Maximal speed, sprint distance, and all volume metrics showed no significant associations with any cognitive change variable (all *ps* > 0.09). Accelerations showed a small, non-significant association with post-match M-VAS (r = 0.175, *p* = 0.079), whereas decelerations were significantly associated with post-match M-VAS (r = 0.241, *p* = 0.015, 95% CI [0.047, 0.416]). High metabolic load distance was also positively associated with post-match M-VAS (r = 0.211, *p* = 0.034), although it was not significantly associated with cognitive change scores.

**Figure 2:**
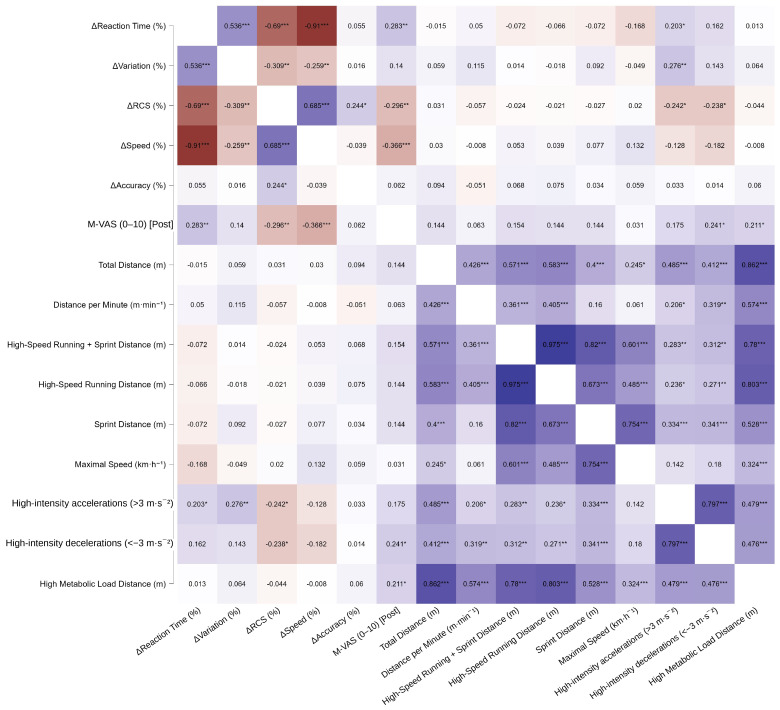
Pearson correlation heatmap of pre- to post-match cognitive change scores, post-match mental fatigue, and GPS-derived external-load metrics (*n* = 101 player–match measurements). Note. Pearson correlation coefficients (r) for *n* = 101 player–match measurements. Blue indicates positive correlations, red indicates negative correlations, and stronger colour intensity represents larger correlation magnitude. Δ variables = percentage change from pre- to post-match; positive Δ for reaction time and variation, negative Δ for RCS, speed, and accuracy indicate poorer post-match performance. M-VAS [Post] = post-match mental fatigue visual analogue scale. HSR = high-speed running. * *p* < 0.05, ** *p* < 0.01, *** *p* < 0.001.

Among cognitive change indices, ΔReaction Time% and ΔVariation% were positively correlated (r = 0.536, *p* < 0.001), and ΔRCS% was negatively correlated with ΔReaction Time% (r = −0.690, *p* < 0.001) and ΔVariation% (r = −0.309, *p* = 0.002), confirming internal consistency across the cognitive battery (Figure 2).

### 3.4. Associations Between Subjective Fatigue, Workload Ratings, and Cognitive Changes

Post-match M-VAS was significantly associated with ΔReaction Time ms (r = 0.281, *p* = 0.004) and ΔRCS% (r = −0.296, *p* = 0.003), and negatively with ΔSpeed% (r = −0.366, *p* < 0.001) and ΔReaction Time% (r = 0.283, *p* = 0.004). Post-match effort (NASA-TLX) correlated with ΔReaction Time ms (r = −0.392, *p* < 0.001), ΔReaction Time% (r = −0.363, *p* < 0.001), and ΔVariation% (r = −0.306, *p* = 0.002). Post-match mental demand was associated with ΔReaction Time ms (r = −0.269, *p* = 0.007) and ΔReaction Time% (r = −0.243, *p* = 0.014) and ΔSpeed% (r = 0.205, *p* = 0.040). Pre-match M-VAS was associated with ΔReaction Time ms (r = 0.265, *p* = 0.007), ΔReaction Time% (r = 0.295, *p* = 0.003), and ΔSpeed% (r = −0.261, *p* = 0.008), and was inversely associated with pre-match motivation (r = −0.405, *p* < 0.001). Pre-match NASA-TLX subscales (mental demand, physical demand, effort, frustration) showed no significant associations with any cognitive change variable (all ps > 0.09).

### 3.5. Effect of Match Outcome on Cognitive Performance Changes

One-way ANOVAs examined whether pre- to post-match cognitive changes differed by match outcome (loss, *n* = 23; draw, *n* = 15; win, *n* = 63). Significant effects emerged for ΔReaction Time% [F(2, 98) = 3.65, *p* = 0.030, η^2^p = 0.069, 90% CI [0.000, 0.174]], ΔSpeed% [F(2, 98) = 3.76, *p* = 0.027, η^2^p = 0.071, 90% CI [0.000, 0.177]], and ΔRCS% [F(2, 98) = 4.94, *p* = 0.009, η^2^p = 0.092, 90% CI [0.007, 0.204]]. Bonferroni-adjusted post hoc tests revealed that losses were associated with greater reaction-time slowing than draws (Δ = 15.71%, 95% CI [1.48, 29.94], d = 0.89, pBonf = 0.025). Speed decrements were also larger following losses than draws (Δ = −11.07%, 95% CI [−20.95, −1.20], d = −0.91, pBonf = 0.022). RCS declined more following losses than draws (Δ = −15.77%, 95% CI [−27.99, −3.54], d = −1.04, pBonf = 0.007). ΔVariation% and ΔAccuracy% did not differ significantly by match outcome (both ps > 0.40; Table 4). These outcome-based effects were re-examined in linear mixed-effects models accounting for repeated measurements within players in Section 3.8.

### 3.6. Effect of Playing Position on Cognitive Performance Changes

Playing position (central defender [CD], *n* = 31; central midfielder [CM], *n* = 21; forward [F], *n* = 18; side defender [SD], *n* = 17; side midfielder [SM], *n* = 14) produced a significant effect on ΔVariation% only [F(4, 96) = 2.53, *p* = 0.045, η^2^p = 0.095, 90% CI [0.000, 0.194]; group means: CD = 49.3%, CM = 42.0%, F = 59.5%, SD = 86.0%, SM = 2.3%]. Bonferroni-corrected post hoc comparisons revealed that side defenders showed significantly greater increases in response inconsistency compared with side midfielders (Δ = 83.8%, 95% CI [6.0, 161.5], d = 1.12 [0.06, 2.18], Bonferroni-adjusted *p* = 0.026). No significant positional effects were found for ΔReaction Time%, ΔAccuracy%, ΔSpeed%, or ΔRCS% (all ps > 0.13). The position-based effect on ΔVariation was also re-examined in the mixed-effects sensitivity analyses in Section 3.8.

### 3.7. Exploratory Predictors of Cognitive Performance Changes: Multiple Regression

Given the repeated player–match structure of the dataset, the following multiple regression analyses were interpreted as exploratory observation-level models. These analyses were conducted to identify candidate predictors of cognitive performance changes before re-examining the same relationships using linear mixed-effects models that accounted for repeated measurements within players. A multiple regression predicting ΔReaction Time (ms) from GPS load metrics (distance·min^−1^, HSR + sprint distance, high-intensity accelerations (>3 m·s^−2^), high-intensity decelerations (<−3 m·s^−2^)), playing position, and match outcome explained 18.1% of the variance [*R*^2^ = 0.181, adjusted *R*^2^ = 0.090, *F*(10, 90) = 1.99, *p* = 0.044]. At the predictor level, playing as a side defender (vs. central defender) was associated with significantly greater reaction time slowing [*B* = 59.46, *SE* = 23.32, *t*(90) = 2.55, *p* = 0.012, 95% CI [13.1, 105.8]], while a draw outcome was associated with less slowing compared with loss [*B* = −75.47, *SE* = 35.15, *t*(90) = −2.15, *p* = 0.034, 95% CI [−145.3, −5.64]]. No GPS metric was a significant individual predictor (all *p*s > 0.07). For the ΔReaction Time model, all variance inflation factors were below 2.50, indicating acceptable multicollinearity.

A second regression predicting ΔVariation% from GPS metrics explained 10.8% of the variance [*R*^2^ = 0.108, adjusted *R*^2^ = 0.070, *F*(4, 96) = 2.90, *p* = 0.026]. High-intensity accelerations (>3 m·s^−2^) were the sole significant predictor [*β* = 0.468, B = 1.212, *SE* = 0.416, *t*(96) = 2.911, *p* = 0.004, 95% CI [0.386, 2.039]; *r* partial = 0.285], indicating that high-intensity acceleration actions uniquely predicted post-match increases in response inconsistency, independent of running volume and deceleration load (Table 5). One potentially influential observation (standardised residual = 3.16, Cook’s *D* = 0.10) was identified but retained, as Cook’s *D* remained below the threshold of 1.0. These regression findings were re-examined using mixed-effects models that explicitly accounted for the within-player correlation structure in Section 3.8.

### 3.8. Sensitivity Analysis: Linear Mixed-Effects Models

Section 3.5, Section 3.6 and Section 3.7 examined cognitive responses using observation-level Δ-scores (post − pre, expressed as percentage change), with each player–match treated as an independent measurement (*n* = 101). This parameterisation isolates pre- to post-match change but does not account for the fact that some players contributed multiple matches. Section 3.8 re-examines the same data using a different analytical framework: linear mixed-effects models on the raw pre- and post-match scores (*n* = 202 measurements from 40 players), with time entered as a fixed factor and player entered as a random intercept. This framework partitions variance into within-player and between-player components, yielding effects that reflect within-player change after between-player differences are absorbed by the random intercept. Consequently, effects that appear in Section 3.5, Section 3.6 and Section 3.7 but not in Section 3.8 indicate that the original signal was carried predominantly by stable between-player differences rather than by within-player match-to-match variation. Effects that appear in Section 3.8 but not in Section 3.5, Section 3.6 and Section 3.7 (e.g., match outcome on accuracy) reflect within-player differences that were obscured in the Δ-score analyses by the inclusion of between-player variance. The two frameworks should therefore be interpreted as complementary rather than redundant.

**Figure 3:**
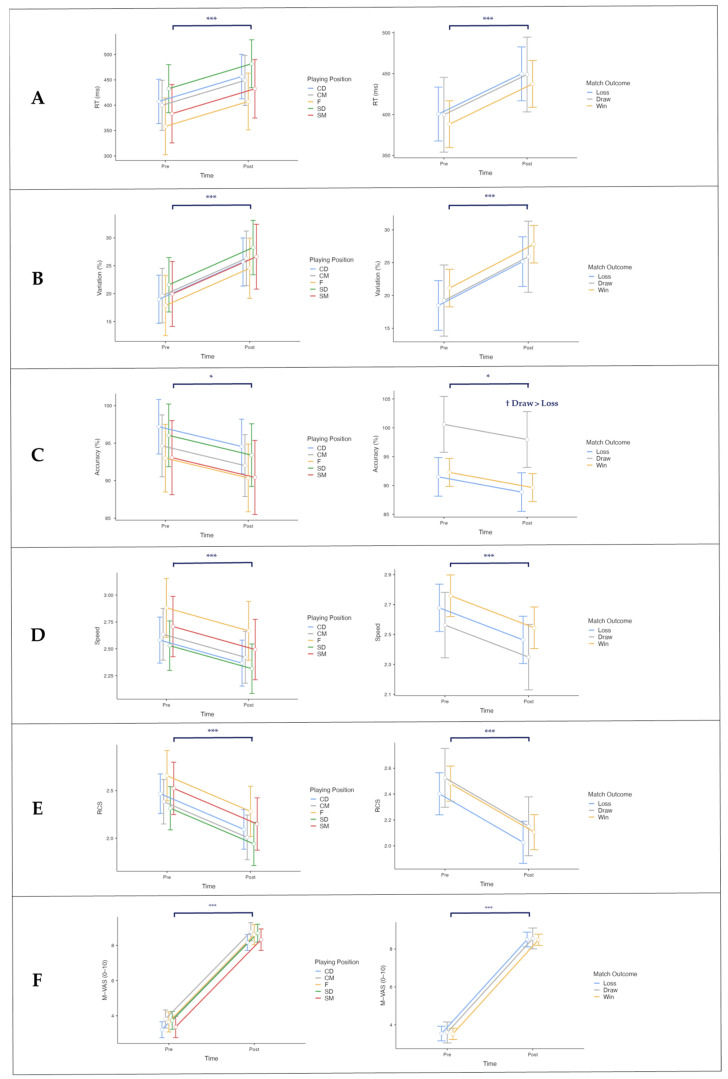
Linear mixed-effects model estimated marginal means for pre- and post-match psychomotor performance and perceived mental fatigue according to playing position and match outcome. Each panel includes two subplots: the left subplot presents estimated marginal means stratified by playing position, whereas the right subplot presents estimated marginal means stratified by match outcome. Panels are presented as follows: (**A**) reaction time; (**B**) response variation; (**C**) accuracy; (**D**) response speed; (**E**) Rate Correct Score; and (**F**) mental fatigue visual analogue scale (M-VAS). Values represent model-derived estimated marginal means from linear mixed-effects models including time, match outcome, and playing position as fixed effects, with a random intercept for player. Error bars indicate 95% confidence intervals. Horizontal brackets with asterisks indicate the omnibus time effect from the mixed-effects models, where * denotes *p* < 0.05 and *** denotes *p* < 0.001. The dagger symbol (†) denotes the significant pairwise match outcome comparison for accuracy, indicating higher accuracy in draws than in losses.

Linear mixed-effects models (Table 6 and Figure 3) with a random intercept for player were fitted to confirm the time effects after accounting for the non-independence of repeated player–match measurements (*n* = 202 measurements from 40 players). All models converged. Intraclass correlation coefficients confirmed substantial between-player variance for reaction time (ICC = 0.60), speed (ICC = 0.64), Rate Correct Score (ICC = 0.51), and Mental Fatigue VAS (ICC = 0.33), with smaller proportions for variation (ICC = 0.28) and accuracy (ICC = 0.19), justifying the inclusion of the random intercept.

The time effect remained statistically significant across all psychomotor outcomes after accounting for player-level dependence and adjustment for GPS-derived load metrics, playing position, and match outcome. Reaction time increased post-match (B = 49.11 ms, *SE* = 6.96, *F*(1, 154.5) = 49.75, *p* < 0.001), as did variation (B = 6.68%, *SE* = 1.11, *F*(1, 161.1) = 36.44, *p* < 0.001) and Mental Fatigue VAS (B = 4.96, *SE* = 0.11, *F*(1, 157.3) = 2079.74, *p* < 0.001). Speed (B = −0.21, *SE* = 0.03, *F*(1, 155.9) = 44.90, *p* < 0.001), Rate Correct Score (B = −0.37, *SE* = 0.04, *F*(1, 155.0) = 95.35, *p* < 0.001), and accuracy (B = −2.63%, *SE* = 1.06, *F*(1, 156.7) = 6.18, *p* = 0.014) decreased post-match. These findings replicate the time effects reported in Section 3.2 and Section 3.1 under a more conservative analytical framework.

In contrast, the omnibus match-outcome effect was no longer significant in the mixed-effects models for reaction time (*F*(2, 108.9) = 0.80, *p* = 0.451), speed (*F*(2, 106.6) = 2.57, *p* = 0.081), Rate Correct Score (*F*(2, 120.6) = 1.02, *p* = 0.362), variation (*F*(2, 145.1) = 1.57, *p* = 0.211), or Mental Fatigue VAS (*F*(2, 138.0) = 0.03, *p* = 0.971), although accuracy retained a significant match-outcome effect (*F*(2, 140.1) = 4.62, *p* = 0.011). Similarly, the omnibus playing position effect was non-significant for all outcomes once between-player variance and load covariates were modelled (all *p*s ≥ 0.302). Among the GPS-derived load metrics, only high-intensity decelerations (<−3 m·s^−2^) reached significance, and only for accuracy (B = 0.088, *SE* = 0.038, *F*(1, 171.0) = 5.28, *p* = 0.023), with all other load metrics non-significant across all outcomes (all *p*s ≥ 0.094). Specifically, the bivariate association between high-intensity accelerations (>3 m·s^−2^) and ΔVariation% reported in Section 3.3 and Section 3.7 did not survive the inclusion of a random intercept for player (B = 0.028, *SE* = 0.040, F(1, 186.3) = 0.49, *p* = 0.484), indicating that the observation-level association was carried predominantly by between-player rather than within-player variance. Marginal *R*^2^ values ranged from 0.11 (accuracy) to 0.88 (M-VAS), and conditional *R*^2^ values from 0.28 (accuracy) to 0.92 (M-VAS), indicating that fixed effects explained a moderate proportion of variance for cognitive outcomes but a substantially larger proportion for subjective fatigue.

These mixed-effects analyses confirm the consistency of the time-related decrements in psychomotor performance and subjective fatigue, while indicating that the previously observed match outcome and playing position effects (Section 3.5, Section 3.6 and Section 3.7) and the contribution of high-intensity accelerations (>3 m·s^−2^) (Section 3.7) were attenuated once within-player dependence was modelled. This pattern suggests that part of the variance attributed to outcome- and position-based group differences in the simpler analyses reflected stable between-player differences rather than acute match-related effects.

## 4. Discussion

The present study examined acute pre- to post-match changes in psychomotor performance, perceived mental fatigue, subjective workload, and their associations with GPS-derived external-load metrics in professional male soccer players. Overall, participation in competitive matches was followed by marked increases in perceived exertion, perceived mental fatigue, mental demand, physical demand, and effort, together with declines across several cognitive-performance indicators. The hypothesis that match play would be associated with slower reaction time, lower cognitive efficiency, and greater response inconsistency was supported. However, the hypothesis that higher external loads—particularly high-speed running and sprint-related metrics—would be broadly associated with cognitive-performance decrements was only partially supported. Among the GPS-derived variables examined, high-intensity accelerations showed small associations with post-match psychomotor performance decrements in the observation-level analyses; however, these associations were weakened and no longer statistically significant in linear mixed-effects models that accounted for within-player dependence, suggesting that part of this signal reflected stable between-player differences rather than acute match-related effects.

### 4.1. Pre- to Post-Match Changes in Perceived Exertion, Mental Fatigue and Workload

The perceptual and workload outcomes indicated that players completed the post-match assessment in a substantially more fatigued psychophysiological state than at baseline. RPE and M-VAS increased markedly, and the NASA-TLX subscales showed large elevations in mental demand, physical demand, and effort. These changes suggest that participation in competitive matches imposed a substantial perceived load, encompassing both physical and mental components. The increase in frustration was statistically significant but comparatively smaller, suggesting that the post-match state was characterised primarily by exertional and demand-related fatigue rather than by a pronounced increase in negative affective response.

The increase in perceived mental fatigue is consistent with the definition of MF as a psychobiological state involving subjective tiredness, reduced energy, and impaired capacity to sustain goal-directed cognitive performance [1]. It is also compatible with psychobiological models proposing that fatigue alters the perceived cost of continued task engagement and may reduce the efficiency with which attention and effort are sustained [1,27]. In the present study, however, motivation did not change significantly from pre- to post-match. This is important because it suggests that the observed post-match cognitive-performance decrements cannot be explained simply by reduced willingness to perform the test. Although motivation was assessed using a brief scale and should not be overinterpreted, the lack of significant decline strengthens the interpretation that post-match decrements reflected an altered fatigue state rather than disengagement from the assessment. This pattern is consistent with evidence from soccer training contexts showing marked increases in M-VAS, RPE, and NASA-TLX scores following cognitively demanding sessions, while motivational indices remained stable [69,70]. Similarly, Rubio-Morales et al. (2022) [71] showed that subjective mental fatigue increased after physical, cognitive, and combined cognitive–physical tasks, with the cognitive and combined protocols producing the largest increases. However, only the cognitive protocol significantly impaired reaction time, supporting the view that subjective mental fatigue and psychomotor performance provide related but non-identical information about fatigue responses.

The simultaneous elevation of physical demand, mental demand, and effort also reinforces the view that fatigue in soccer is multifactorial. Competitive match play requires sustained locomotor output, repeated high-intensity actions, and continuous perceptual-cognitive regulation under tactical and temporal constraints. This aligns with contemporary football fatigue frameworks, which emphasise that physical expressions of fatigue occur in parallel with perceptual, psychological, and cognitive demands [25,26]. This multidimensional fatigue profile has been documented in ecologically valid soccer contexts, where M-VAS and NASA-TLX subscales were jointly elevated following real training sessions with increased tactical complexity [70,72] and across training days closest to match-day within a competitive microcycle, with the first training session after a match (MD + 1) consistently showing the highest mental fatigue values [73]. The present findings therefore support the use of both subjective and objective indicators when evaluating post-match readiness.

### 4.2. Pre- to Post-Match Changes in Cognitive Performance

Cognitive performance deteriorated significantly after match play across reaction time, accuracy, speed, and response variability. Reaction time slowing indicates that players responded less rapidly to visual stimuli after the match, while the reduction in accuracy indicates a modest but significant decline in response quality. The decrease in speed and increase in variation further suggest that the post-match cognitive state was not limited to general slowing but involved reduced efficiency and less stable responding.

This multidimensional pattern is relevant because reaction time should not be interpreted in isolation. A slower response may reflect fatigue-related impairment, but it may also reflect a deliberate shift toward accuracy. In the present findings, however, slower reaction time occurred alongside lower accuracy and greater response variability. This reduces the likelihood that the post-match pattern reflects a simple speed–accuracy trade-off and instead suggests a broader reduction in cognitive efficiency [74,75]. The decline in RCS-related performance is particularly informative because RCS integrates speed and accuracy and is intended to capture the rate of correct responding rather than one dimension of performance alone [75]. In applied sport settings, faster responses are practically meaningful only when they are accompanied by preserved accuracy and stable response consistency. This is important because speed and accuracy are interdependent, and reaction-time values vary according to task complexity, stimulus–response demands, individual baseline, athletic population, and the specific perceptual–motor requirements of the task [74,75,76,77,78].

The increase in response variability may be especially meaningful in the context of mental fatigue. Greater intra-individual variability reflects less consistent responding across trials and has been associated with attentional instability, lapses, and reduced cognitive control [79,80,81]. In soccer, where successful performance depends on repeated rapid perception–action cycles, reduced response stability may be practically relevant even when the absolute task is relatively simple. The present PFTT does not reproduce soccer-specific tactical decision-making, but it provides a brief index of psychomotor readiness that may be sensitive to the acute demands of competition. From a neurophysiological perspective, the present pattern may reflect transient disruption in the neural systems supporting sustained attention, inhibitory control, and effort regulation. Psychobiological models suggest that mental fatigue alters the perceived cost of continued task engagement, which may reduce the efficiency of prefrontal and cingulate control processes involved in maintaining goal-directed performance [1,2,27]. In soccer, these mechanisms are particularly relevant because players must repeatedly regulate attention, inhibit premature responses, and update decisions under time pressure and changing tactical constraints [4,6]. The observed post-match slowing, lower accuracy, and increased response variability are therefore consistent with a temporary reduction in psychomotor control rather than a simple loss of motivation, especially because motivation did not decline significantly.

These results are consistent with previous literature showing that MF can impair sustained attention, psychomotor vigilance, executive control, and sport-specific performance [2,4,6]. Experimental soccer studies have reported that mentally fatiguing tasks impair soccer-specific decision-making speed and accuracy and can reduce physical or technical performance in subsequent exercise contexts [6,8]. The systematic review by Gonzalez-Villora et al. [82] confirmed that perceptual-cognitive performance is the soccer domain most consistently impaired by MF across study designs, with decision-making speed and accuracy representing the outcomes most sensitive to fatigue-induced deterioration. Furthermore, Kunrath et al. [83] demonstrated in soccer-specific small-sided games that MF reduced peripheral perception, slowed response times, and restricted decision-making accuracy, while gross physical output measured via GPS was largely preserved—a dissociation that aligns with the present pattern of marked cognitive decrements in the relative absence of extreme external-load differences. The present study extends this literature by showing that cognitive-performance decrements can be detected immediately after competitive matches, rather than only following laboratory-induced mental fatigue protocols.

### 4.3. Associations Between GPS-Derived External Load and Cognitive Performance Changes

The associations between GPS-derived external load and cognitive change only partially supported the study hypothesis. Contrary to expectations, total distance, distance per minute, high-speed running plus sprint distance, high metabolic load distance, maximal speed, and sprint distance were not significantly associated with any cognitive change variable. These null findings suggest that post-match cognitive-performance decrements were not simply proportional to global locomotor volume or high-speed running exposure.

The most consistent association was observed for high-intensity accelerations (>3 m·s^−2^). Greater high-intensity acceleration load was associated with larger increases in reaction time, greater increases in response variability, and greater reductions in RCS. This pattern suggests that acceleration-intensive match profiles may be more closely related to post-match psychomotor instability than distance- or speed-based metrics. However, these associations should be interpreted cautiously. When within-player dependence was modelled using linear mixed-effects analyses, the associations between high-intensity accelerations (>3 m·s^−2^) and cognitive outcomes were attenuated and no longer reached statistical significance, while high-intensity decelerations (<−3 m·s^−2^) retained a significant association only with accuracy. This pattern suggests that the bivariate acceleration–cognition associations may have partly reflected stable between-player tendencies, whereby certain players who routinely accumulated more high-intensity actions also showed greater post-match cognitive change, rather than a robust within-player coupling between acceleration load and acute psychomotor-performance decrements.

One possible interpretation is that high-intensity accelerations represent actions embedded within tactically demanding phases of play, such as pressing, counter-pressing, defensive recovery, duels, and rapid transitions. These actions may impose simultaneous mechanical, perceptual, and decision-making demands that are not fully captured by high-speed distance alone. This interpretation finds support in training-based evidence showing that task conditions which increase tactical and cognitive complexity—such as modified scoring rules [84] and decision-making constraints [70]—simultaneously elevate both subjective mental fatigue and high-intensity locomotive demands, including accelerations, while HSR and sprint metrics remain comparatively less affected. Similarly, Ponce-Bordón et al. [85] reported that training-task orientations imposing higher cognitive load were associated with greater mechanical output, consistent with the view that cognitively demanding actions cluster with high-intensity accelerative efforts.

This interpretation is consistent with match-analysis literature indicating that accelerations and decelerations contribute substantially to the mechanical and metabolic demands of soccer, even when they occur below traditional high-speed running thresholds [15,16]. The present findings suggest that such actions may also be relevant for understanding acute cognitive readiness. Nevertheless, this explanation remains cautious. The study did not include video-coded tactical context or event-level analysis, so it cannot determine whether accelerations were cognitively demanding because of their mechanical characteristics, their tactical context, or both.

The weaker findings for decelerations also deserve attention. High-intensity decelerations (<−3 m·s^−2^) were associated with poorer ΔRCS% and with higher post-match M-VAS, but their associations with reaction time and variation did not reach significance, and they were not independent predictors in the regression model. This suggests that deceleration load may contribute to perceived or composite fatigue responses, but its relationship with specific psychomotor outcomes was weaker than that of acceleration load. Notably, in the linear mixed-effects analyses (Section 3.8), high-intensity decelerations were the only GPS-derived load metric that retained a significant fixed-effect association with any cognitive outcome, specifically post-match accuracy. However, this finding should not be interpreted as evidence of a direct mechanistic pathway, because the study was observational, the association was modest, and the fixed effects explained only a limited proportion of variance in accuracy (marginal *R*^2^ = 0.11). Therefore, larger samples and more context-sensitive modelling may further clarify the independent and potentially overlapping contributions of acceleration and deceleration demands to post-match psychomotor responses.

Overall, these findings indicate that, at the player–match observation level, cognitive fatigue-related changes after match play were more closely associated with high-intensity acceleration and deceleration actions than with total running output, although these associations were attenuated when within-player dependence was modelled. This pattern may suggest that the qualitative nature of high-intensity mechanical actions may carry information about cognitive readiness beyond locomotor volume, but that this association is partly carried by stable between-player differences rather than within-player match-to-match variation.

### 4.4. Associations Between Subjective Fatigue, Workload Ratings, and Cognitive Changes

The associations between subjective fatigue, workload ratings, and cognitive changes were meaningful but not entirely uniform. Post-match M-VAS was associated with greater reaction-time slowing and lower RCS-related performance, supporting the interpretation that subjective mental fatigue was linked to poorer psychomotor efficiency. This is consistent with psychobiological accounts in which increased perceived fatigue and effort are related to a reduced capacity to sustain efficient performance [1,27,86]. However, subjective and objective fatigue indicators should not be considered interchangeable. Rubio-Morales et al. [71] reported that cognitive and combined cognitive–physical protocols produced the largest increases in subjective mental fatigue, although only the cognitive protocol significantly impaired reaction time. Similarly, García-Calvo et al. [70] reported that NASA-TLX mental-demand scores covaried with changes in external physical output in constrained soccer training tasks. Together, these findings support the use of subjective fatigue ratings and objective psychomotor outcomes as complementary, rather than redundant, indicators of fatigue responses.

Pre-match M-VAS was also associated with subsequent decrements in reaction time and speed. This finding may suggest that players who began the match with higher perceived mental fatigue were more vulnerable to post-match cognitive decline. Such an interpretation is consistent with the view that baseline cognitive state may influence how athletes tolerate subsequent physical and cognitive demands. In a microcycle monitoring study of semiprofessional soccer players, Díaz-García et al. [73] showed that higher mental fatigue on days closer to the match was associated with altered training responses, suggesting that pre-competition MF level is a meaningful moderator of subsequent cognitive and physical performance. However, the observational design does not allow this to be interpreted causally. Pre-match M-VAS may reflect prior sleep, recovery, emotional state, accumulated training load, or other unmeasured factors.

The negative association between pre-match M-VAS and pre-match motivation is coherent with the conceptualisation of MF as a state involving reduced energy and altered willingness to invest effort [1]. However, because motivation did not change significantly after the match, the post-match decline in cognitive performance should not be reduced to motivational loss. Rather, the results suggest that perceived fatigue, motivation, and psychomotor performance are related but distinct components of the broader readiness profile.

Some NASA-TLX associations require more cautious interpretation. Post-match effort and mental demand showed significant associations with reaction-time and speed-related outcomes, but the direction of some relationships was not entirely consistent with a simple model in which higher workload always corresponds to greater impairment. This may reflect individual differences in rating behaviour, positional role, match involvement, perceived contribution, or emotional response to match outcome. It may also indicate that subjective workload and objective cognitive performance do not measure the same construct. Accordingly, subjective ratings may be interpreted as complementary to, rather than substitutes for, objective psychomotor outcomes.

An additional pattern emerged within the GPS–subjective fatigue associations: among the locomotor-volume metrics, only high metabolic load distance (HMLD) was significantly associated with post-match M-VAS (r = 0.211, *p* = 0.034). Total distance, distance per minute, HSR plus sprint distance, sprint distance, and maximal speed were not (all rs ≤ 0.175, all ps ≥ 0.063). HMLD weights distance accumulated under elevated metabolic demand, including accelerations, decelerations, and changes in direction, rather than locomotor volume alone. The selective association with subjective fatigue—in the absence of any association with the objective psychomotor outcomes—suggests that perceived mental fatigue may track the mechanically demanding portion of locomotor output more closely than overall running volume, even when objective psychomotor change does not. This is coherent with the parallel pattern observed for high-intensity decelerations, which were associated with post-match M-VAS (r = 0.241, *p* = 0.015) as well as with ΔRCS%, and reinforces the interpretation that the mechanical, rather than the locomotor-volume, component of match load is the aspect most relevant for subjective fatigue.

### 4.5. Effect of Match Outcome on Cognitive Performance Changes

Match outcome was associated with selected cognitive change scores, with losses producing greater reaction-time slowing and larger reductions in speed and RCS than draws. This pattern may reflect the broader competitive demands associated with unsuccessful match outcomes, including greater tactical disruption, defensive pressure, and reduced control of play. However, the omnibus match-outcome effect did not reach statistical significance in the linear mixed-effects models for reaction time, speed, or Rate Correct Score (Section 3.8), although a within-player effect of match outcome on accuracy was revealed only in the linear mixed-effects model (F(2, 140.1) = 4.62, *p* = 0.011; Section 3.8), where post-match accuracy was higher after draws than after losses. This effect was absent in the observation-level ANOVA (Section 3.5; *p* = 0.597) and therefore reflects within-player rather than between-player variation, indicating that Δ-score analyses obscured a genuine within-player coupling between match outcome and post-match response correctness. This indicates that the loss-related cognitive disadvantage observed in the observation-level analyses was substantially attenuated once between-player variance was modelled, and the interpretation that competitive context modulates post-match psychomotor performance should therefore be treated as preliminary.

Recent match-analysis evidence from elite international competition indicates that match outcome is associated with contextual performance indicators such as total distance covered, defensive pressure, possession, and attempts at goal [87], suggesting that a loss may reflect a broader tactical and physical context rather than a simple categorical result. In the present study, losses were associated with greater psychomotor decrements in observation-level analyses, but these effects were largely attenuated after accounting for within-player dependence. Therefore, although high-intensity accelerations may occur during tactically demanding phases such as pressing, defensive recovery, and transitions, the present data cannot determine whether acceleration-intensive demands contributed directly to the stronger decrements observed after losses.

These findings suggest that the competitive context of the match may be relevant for post-match cognitive readiness. A loss may be accompanied by greater emotional load, perceived pressure, frustration, tactical disruption, or sustained defensive and transitional demands, all of which could contribute to poorer post-match cognitive efficiency. These results are consistent with microcycle monitoring evidence demonstrating that the first training session after a match (MD + 1) consistently shows the highest mental fatigue ratings across the week, and that these values are modulated by the competitive demands of the preceding match [73]. While that study compared regular-season and play-off contexts rather than individual match outcomes, its finding that more demanding competitive periods amplify post-match MF supports the interpretation that losses—which may impose greater tactical, physical, and psychological demands—produce more pronounced cognitive disruption than draws.

However, the interpretation of match outcome must remain cautious. Outcome is not an isolated exposure and cannot be treated as a direct cause of cognitive decline. Losing may reflect stronger opposition, greater match difficulty, unfavourable scoreline evolution, tactical instability, or higher psychological stress. It is also possible that players in lost matches experienced different physical or decision-making demands that were not fully captured by the GPS variables included in the analyses. Therefore, the present findings indicate an association between match outcome and cognitive change, not a causal effect of losing.

The absence of significant outcome effects for ΔVariation% and ΔAccuracy% is also informative. Match outcome was not significantly associated with response variability at either the observation level or in the linear mixed-effects model, suggesting that response inconsistency was driven more by individual and load-related factors than by competitive outcome. In contrast, post-match accuracy emerged in the linear mixed-effects analysis (Section 3.8) as the only outcome where both match outcome and high-intensity decelerations retained significant fixed-effect associations—associations that were not apparent in the corresponding observation-level analyses. The convergence of two mixed-effects-only fixed effects on the same outcome suggests that post-match accuracy may be the cognitive dimension most responsive to within-player variation in match context. The direction of the match-outcome effect at the mixed-effects level should, however, be confirmed by reporting the corresponding pairwise contrasts, as the observation-level post hoc comparisons on ΔAccuracy% did not reach significance.

### 4.6. Effect of Playing Position on Cognitive Performance Changes

Playing position was only associated with ΔVariation%, with side defenders showing greater increases in response inconsistency than side midfielders. In contrast, positional effects were not significant for reaction time, accuracy, speed, or RCS. This selective finding tentatively suggests that positional role may influence the stability of post-match psychomotor performance more than mean response speed or accuracy. However, this position-based difference was not retained in the linear mixed-effects analysis of response variability (Section 3.8), suggesting that the initial contrast was weakened once between-player variance was explicitly modelled. Accordingly, side defenders and side midfielders may differ in stable individual characteristics that co-vary with response variability, rather than the side-defender role itself causing greater post-match response inconsistency.

The positional pattern should be interpreted with restraint because group sizes were relatively small, and the analysis was based on nominal playing position rather than detailed tactical role or match-specific responsibilities. Nevertheless, the finding is plausible within the context of soccer match demands. Wide players, including side defenders and side midfielders, often perform repeated high-intensity runs, recovery actions, overlaps, defensive duels, and transitions. Previous match-analysis work has shown that external-load demands differ by position, with wide roles often exposed to substantial high-speed and sprint demands [13,14].

Previous match-analysis work has shown that external-load demands differ according to playing position, tactical role, and match context, with wide players often exposed to substantial high-speed and sprint demands [13,14]. Importantly, positional differences in running profile extend beyond simple speed-zone metrics: for example, in elite youth football competing in a 1-4-3-3 formation, Michailidis et al. [54] showed that while central midfielders accumulated the greatest total distance, side players were characterised by elevated high-intensity running, sprinting, and maximal-speed efforts—a profile associated with repeated acceleration-deceleration cycles rather than sustained locomotion. At the highest competitive level, Modric et al. [88] demonstrated that position-specific running demands in UEFA Champions League play are further shaped by phase of play, with wide defensive roles exposed to particularly demanding recovery and transition sequences. These findings are relevant to the present results because high-intensity accelerations—not HSR or sprint distance—were the GPS metric most strongly associated with post-match cognitive-performance decrements. If side defenders accumulate more of these tactically embedded acceleration efforts than side midfielders, as the positional-load literature implies, this may partly explain why side defenders showed greater post-match response inconsistency.

The present findings suggest that such positional demands may also be associated with variation in post-match cognitive stability. This extends microcycle monitoring research in which playing time—itself partly position-dependent—was identified as a significant moderator of post-match mental fatigue, with players who accumulated more minutes showing greater MF elevations [73]. If position-specific workload moderates MF accumulation during training, it is plausible that analogous position-specific effects operate during matches. Although previous reviews have highlighted the need for further work on behavioural and cognitive-performance responses in soccer-specific fatigue contexts [82], no study, to our knowledge, has directly compared pre- to post-match psychomotor performance changes across playing positions in professional soccer. The present positional contrast is therefore an early observation that requires confirmation in larger, well-powered samples permitting reliable mixed-effects modelling.

The difference between side defenders and side midfielders is not straightforward. Both roles may involve substantial wide-channel activity, but their tactical constraints differ. Side defenders may face repeated defensive recovery actions, one-versus-one duels, and transitions from defensive to attacking phases, whereas side midfielders may have different offensive and pressing responsibilities depending on the team structure. Because these contextual demands were not coded, the present data cannot explain why side defenders showed greater response inconsistency. Future work should combine positional classification with tactical event coding and individual external-load profiles to determine whether the observed positional effect reflects role-specific match demands or sample-specific variation.

### 4.7. Exploratory Predictors of Cognitive Performance Changes: Multiple Regression

The regression analyses provide a more integrated view of the predictors of post-match cognitive change. The model predicting ΔReaction Time explained 18.1% of the variance, although the adjusted *R*^2^ was lower, indicating that the explanatory value of the model was modest. Within this model, side-defender status was associated with greater reaction-time slowing relative to central defenders, while draw outcome was associated with less slowing compared with loss. No GPS metric independently predicted ΔReaction Time.

This finding suggests that reaction-time change may be influenced by contextual or role-related factors not fully captured by the selected GPS variables. It also reinforces the need for caution when interpreting simple bivariate associations particularly in applied sport datasets where external load, tactical role, match context, and individual player characteristics are closely interrelated [18,25,26]. Although accelerations were correlated with ΔReaction Time%, they did not independently predict reaction-time change once other predictors were included. Reaction time may therefore be sensitive to a broader combination of positional, contextual, psychological, and physiological influences.

The ΔVariation% regression model was more specific. Although it explained a modest proportion of variance, high-intensity accelerations (>3 m·s^−2^) emerged as the sole significant independent predictor in the observation-level regression. However, this association did not survive the inclusion of a random intercept for player in the linear mixed-effects model (B = 0.028, *SE* = 0.040, F(1, 186.3) = 0.49, *p* = 0.484; Section 3.8), indicating that the acceleration–variability relationship observed at the observation level was carried predominantly by between-player rather than within-player variance. The regression result therefore suggests that players who tended to accumulate more high-intensity accelerations (>3 m·s^−2^) across matches also tended to show greater response inconsistency, but does not support a within-player coupling in which any given player’s acceleration load in a specific match predicts their cognitive change in that same match. This supports a cautious interpretation that acceleration load may be selectively associated with response inconsistency at the player–match observation level, but not necessarily as a within-player predictor of acute psychomotor change. The finding is theoretically coherent because response variability may be especially sensitive to fluctuations in attentional control, task engagement, and sustained cognitive control [79,80,81].

Still, the predictive strength should not be overstated. The model explained 10.8% of the variance in ΔVariation%, meaning that most of the variability in post-match response inconsistency remained unexplained. This is expected in an applied match setting, where cognitive readiness is likely influenced by multiple interacting factors, including sleep, recovery, tactical role, emotional state, match context, previous load, and individual resilience. Such multifactorial interpretation is consistent with contemporary perspectives on football fatigue and athlete monitoring, which emphasise that performance readiness cannot be inferred from external-load metrics alone [5,18,25,26]. The retained influential observation also suggests that individual match responses may vary substantially, although Cook’s distance did not indicate a severe distortion of the model.

Taken together, the observation-level regression results indicated that GPS-derived acceleration load, positional role, and match outcome each provided partial information about post-match cognitive change. However, none of these predictors retained a significant within-player effect when the data were re-analysed using mixed-effects models (Section 3.8), with the limited exception of decelerations on accuracy. The findings therefore support a multidimensional monitoring framework in which external load, subjective fatigue, contextual variables, and objective cognitive outcomes are interpreted together [18,25,26], while underscoring that the degree to which any individual GPS metric tracks acute within-player cognitive change appears smaller than the bivariate associations would suggest.

### 4.8. Practical Implications

Within the constraints of the present observational design, the findings suggest that brief psychomotor assessments may be useful for monitoring acute cognitive readiness before and after competitive soccer matches. However, practitioners should avoid interpreting reaction time in isolation. In the present study, post-match cognitive decrements were expressed through slower reaction time, reduced accuracy, lower speed–accuracy efficiency, and greater response variability. Therefore, a multidimensional cognitive profile appears more informative than any single outcome. In applied settings, short assessments that combine reaction time, accuracy, response consistency, and an integrated speed–accuracy index may provide a more complete indication of post-match psychomotor status.

A practical implication is that cognitive monitoring should be interpreted relative to each player’s own baseline. Because the mixed-effects models showed substantial between-player variance, absolute comparisons between players may be misleading. For example, one player may consistently present slower reaction times but remain stable from pre- to post-match, whereas another may show a large post-match decrement relative to his usual profile. Practitioners should therefore prioritise within-player changes across repeated matches rather than relying only on team-level averages or single post-match values.

The findings also indicate that GPS-derived running outputs should not be used as standalone indicators of acute cognitive readiness. Total distance, high-speed running, sprint distance, and maximal speed were not significantly associated with cognitive-performance changes. These metrics remain essential for external-load monitoring, but they do not appear sufficient to identify post-match psychomotor impairment. Consequently, players who complete high running volumes or sprint distances should not automatically be assumed to have the greatest cognitive decrement. Conversely, players with moderate external loads may still show meaningful post-match slowing, reduced accuracy, or increased response variability.

Acceleration and deceleration load may still have applied relevance when interpreted alongside cognitive data. Although the bivariate associations between high-intensity accelerations and response variability were weakened in the mixed-effects models (Section 3.8), players who repeatedly combine high acceleration/deceleration loads with poorer post-match response consistency may warrant closer monitoring. In practice, this could inform recovery planning, subsequent training prescription, or the timing of cognitively demanding tactical sessions. For example, when a player presents both high neuromuscular load and marked post-match psychomotor deterioration, coaches may consider reducing additional high-intensity or decision-heavy work in the following session.

In practice, a loss may function as a contextual warning signal rather than a causal explanation. When players experience a loss and also show slower reaction time, reduced speed–accuracy efficiency, or higher perceived mental fatigue, practitioners should consider that the player may require closer recovery monitoring before the next training exposure. This does not mean that all players are cognitively fatigued after losing, but rather that post-loss assessments may help identify individuals who present reduced readiness for cognitively demanding tactical work, video analysis, or high-pressure decision-making drills.

The association between higher pre-match M-VAS and greater post-match cognitive-performance decrement is particularly relevant for practice. It suggests that players who begin a match with elevated perceived mental fatigue may be more vulnerable to subsequent psychomotor decline. Therefore, pre-match cognitive state should be considered part of the monitoring process, not only post-match fatigue. When players report elevated pre-match mental fatigue, practitioners may consider practical strategies such as reducing unnecessary cognitive load before kick-off, simplifying final tactical instructions, limiting distracting screen-based activity, and providing a quieter preparation environment. Brief cognitive-readiness or cognitive-priming routines may also be explored, although their use in competitive soccer requires further evidence.

Overall, the present findings support an integrated approach to fatigue monitoring in professional soccer. Fatigue should not be treated as a purely physical construct, because match play requires continuous interaction between physical actions, perception, inhibition, rapid information processing, and decision-making. A practical monitoring framework should therefore combine GPS-derived mechanical demands, subjective measures of perceived exertion and mental fatigue, and brief objective psychomotor assessments. Such an approach may help practitioners identify players who require closer recovery monitoring, modified training exposure, or additional support before returning to high cognitive and physical demands.

### 4.9. Limitations

Several limitations should be considered. First, the observational repeated-measures design does not allow causal interpretation. External load was not experimentally manipulated; therefore, associations between acceleration load, match outcome, playing position, and cognitive change may reflect contextual match demands rather than direct effects. Second, data collection was conducted in an applied professional soccer environment, where post-match assessment is challenging. The brief mobile app-based protocol improved feasibility and compliance but limited the range of cognitive functions assessed. Third, the dataset included 40 players and 101 player–match measurements, meaning that some players contributed data from more than one match. Only measurements from outfield players who completed at least 60 min of match play were retained for the analyses, whereas goalkeepers were excluded. Both absolute and relative external-load indicators, including distance·min^−1^, were used to address unequal exposure. However, separate comparisons between players completing 60 min and those completing the full match or 90+ minutes were not conducted; therefore, possible differences in fatigue responses according to match exposure duration cannot be excluded. This repeated-monitoring structure improves ecological relevance but introduces within-player dependence, which was addressed using linear mixed-effects sensitivity analyses (Section 3.8). Nevertheless, potential match-level clustering was not fully modelled because a random intercept for match was not included, due to the limited number of matches and the risk of unstable match-level variance estimation. The matches-per-player distribution was unbalanced (median = 2 matches, range = 1–8), with half the sample contributing a single match; this skew, combined with the moderate-to-high intraclass correlations observed for several outcomes (range = 0.19–0.64), reduces the effective sample size for fixed-effect estimation in the mixed-effects models. The non-significant match outcome and playing position effects in Section 3.8 should therefore be interpreted as inconclusive rather than as positive evidence of true null effects.

Fourth, the sample was limited to male players from a professional team competing in a national-level championship, which may restrict generalisability to lower competitive levels and teams with different tactical or competitive profiles. Fifth, the 90 s PFTT provides feasible field-based indices of psychomotor readiness, but, given its brief 25-stimulus structure and limited ecological specificity, the findings should be interpreted as brief psychomotor-performance indicators rather than direct evidence of impaired match decision-making, tactical cognition, executive function, or soccer-specific cognitive fatigue. Sixth, the absence of a non-match control condition means that repeated testing, time of day, travel demands, pre-match arousal, post-match emotional state, and other contextual factors cannot be fully separated from the effects of match play. Seventh, several additional potential confounders were not collected or controlled, including sleep quality, fixture congestion in the days preceding the match, academic or personal stress, nutrition and hydration status, prior training load, recovery status, opponent strength, tactical formation, scoreline evolution, and environmental conditions. Finally, multiple statistical comparisons were conducted, increasing the possibility of Type I error, while the modest player-level sample size may have increased the possibility of Type II error. Therefore, the findings should be interpreted as preliminary evidence of selective associations rather than definitive predictive models.

### 4.10. Future Research

Future research should extend the present mixed-effects approach by using larger samples and more balanced repeated-measures designs that permit reliable estimation of within-player slopes for external-load, outcome, and position effects. The unbalanced contribution of matches per player in the present sample limited the power to detect within-player relationships in the mixed-effects analyses; well-powered replications would clarify whether the acceleration–variability association observed in the bivariate analyses reflects a consistent within-player relationship or stable between-player differences.

Future studies should also combine GPS data with video-coded tactical context, scanning behaviour, heart rate responses, heart rate variability, and objective indicators of tactical decision-making, such as pass selection, pass timing, pass accuracy, and decision appropriateness. This approach would help determine whether acceleration-related psychomotor changes are attributable mainly to mechanical load, perceptual-decision demands, autonomic regulation, recovery status, or the interaction among these factors. The inclusion of HRV may be particularly relevant, as recent evidence in U19 soccer players suggests that RMSSD and LnRMSSD are associated with subjective recovery indicators, including sleep quality, mental energy, fatigue, and previous-day RPE [89]. Brief mobile psychomotor tasks should be compared with soccer-specific decision-making, anticipation, visual-search, and inhibitory-control tasks to clarify their relevance for match performance. Future work should examine whether subjective, behavioural, physiological, neuromuscular, sleep, and neurocognitive markers identify the same fatigue state or capture different components of post-match readiness. Match-outcome effects should be examined alongside contextual variables such as opponent strength, scoreline progression, match location, competitive importance, tactical formation, substitutions, playing time, and individual performance ratings. Future longitudinal studies could examine whether post-match changes in reaction time, response variability, or RCS add information to injury-surveillance, load-monitoring, or return-to-play models. Future studies should also examine whether individualised thresholds or deviations from baseline can identify practically relevant profiles of cognitive fatigue or incomplete recovery. In addition, intervention studies could examine whether cognitive training, brain endurance training [43], brief pre-event cognitive priming protocols [47], and environmental, nutritional, and recovery modalities [90,91,92,93] improve tolerance to match-induced psychomotor fatigue and reduce post-match decrements in reaction time, response variability, or RCS. Finally, future studies should include larger and more diverse samples, including female players, youth players, lower-level professionals, injured or recently returned players, and teams with different tactical systems.

## 5. Conclusions

Competitive soccer match play was associated with substantial increases in perceived exertion, perceived mental fatigue, and subjective workload, alongside decrements in brief psychomotor performance. These post-match changes were confirmed in linear mixed-effects analyses that accounted for within-player dependence. In the initial player–match-level analyses, high-intensity accelerations (>3 m·s^−2^) were associated with greater reaction-time slowing, increased response variability, and reduced psychomotor efficiency, suggesting a possible relationship between acceleration-intensive match profiles and post-match psychomotor disturbance. However, these associations were attenuated and no longer reached statistical significance when repeated measurements within players were modelled, indicating that they should be interpreted as exploratory player–match-level associations rather than reliable within-player markers of acute mental fatigue or psychomotor decline. In contrast, high-intensity decelerations (<−3 m·s^−2^) retained a limited fixed-effect association with accuracy only. Total distance, high-speed running, and sprint distance were not meaningfully associated with psychomotor change in either analytical framework, while high metabolic load distance showed only a small player–match-level association with post-match perceived mental fatigue and was not associated with psychomotor performance changes. Acceleration- and deceleration-related demands may therefore provide useful contextual information when interpreting post-match cognitive readiness, but their role remains preliminary and requires confirmation in larger samples using more complete multilevel designs. Because the study was observational, involved unbalanced repeated player–match measurements, and did not include a non-match control condition, the findings should be interpreted as preliminary evidence rather than causal proof that GPS-derived mechanical actions determine acute mental fatigue or psychomotor decline.

## Figures and Tables

**Table 1:** Pre- to post-match cognitive performance (*n* = 101).

Variable	Pre *M* (*SD*)	Post *M* (*SD*)	Δ [95% CI]	Cohen’s *d* [95% CI]
Reaction Time (ms) ↑^a^	389.7 (61.5)	438.9 (94.1)	+49.1 [34.4, 63.8] ***	−0.62 [−0.82, −0.41]
Accuracy (%) ↓^a^	93.7 (7.3)	91.1 (9.3)	−2.63 [−4.44, −0.83] **	0.32 [0.10, 0.54]
Speed ↓^a^	2.73 (0.41)	2.51 (0.39)	−0.21 [−0.28, −0.15] ***	0.53 [0.35, 0.71]
Variation (%) ↑^a^	19.8 (7.6)	26.5 (10.7)	+6.68 [4.17, 9.20] ***	−0.72 [−1.01, −0.43]
RPE (0–10) ↑	1.47 (0.61)	8.95 (0.79)	+7.49 [7.28, 7.69] ***	−10.59 [−12.11, −9.08]

Note. ↑ = increased post-match; ↓ = decreased post-match. ᵃ Cognitive performance decline: higher reaction time and higher variation reflect worse performance; lower accuracy and lower speed reflect worse performance. Δ = post minus pre. Cohen’s negative d indicates post > pre. All variables shown in Table 1 changed significantly from pre- to post-match. ** *p* < 0.01, *** *p* < 0.001.

**Table 2:** Pre- to post-match changes in subjective fatigue, rating of perceived exertion, and workload (*n* = 101).

Measure	Pre *M* (*SD*)	Post *M* (*SD*)	Δ [95% CI]	Cohen’s *d* [95% CI]
Mental Demand	38.1 (9.0)	83.0 (10.5)	+44.9 [42.3, 47.4] ***	−4.57 [−5.27, −3.88]
Physical Demand	31.3 (9.5)	88.7 (8.6)	+57.4 [55.0, 59.9] ***	−6.37 [−7.30, −5.43]
Effort	30.8 (10.3)	85.8 (9.9)	+55.1 [52.3, 57.8] ***	−5.45 [−6.26, −4.64]
Frustration	25.1 (8.4)	29.5 (11.7)	+4.46 [2.41, 6.50] ***	−0.44 [−0.65, −0.23]
Motivation (0–4)	3.76 (0.45)	3.67 (0.55)	−0.09 [−0.20, 0.02]	0.16 [−0.04, 0.36]
M-VAS (0–10)	3.52 (0.91)	8.48 (0.96)	+4.96 [4.73, 5.19] ***	−5.31 [−6.10, −4.53]
RPE (0–10)	1.47 (0.61)	8.95 (0.79)	+7.49 [7.28, 7.69] ***	−10.59 [−12.11, −9.08]

Note. NASA-TLX subscales: mental demand, physical demand, effort, frustration (0–100 each). M-VAS = mental fatigue visual analogue scale (0–10). RPE = rating of perceived exertion (0–10; higher = greater perceived exertion). Δ = post minus pre. *** *p* < 0.001.

**Table 3:** Pearson correlations between GPS external load metrics, cognitive change scores, and post-match mental fatigue.

GPS Metric	ΔRT%	ΔRCS%	ΔVariation%	ΔAccuracy%	M-VAS [Post]
High-intensity accelerations (>3 m·s^−2^)	0.203 *	−0.242 *	0.276 **	0.033	0.175
High-intensity decelerations (<−3 m·s^−2^)	0.162	−0.238 *	0.143	0.014	0.241 *
Total Distance (m)	−0.015	0.031	0.059	0.094	0.144
Distance per minute (m·min^−1^)	0.050	−0.057	0.115	−0.051	0.063
HSR + Sprint Distance (m)	−0.072	−0.024	0.014	0.068	0.154
High Metabolic Load Distance (m)	0.013	−0.044	0.064	0.060	0.211 *
Maximal Speed (km·h^−1^)	−0.168	0.020	−0.049	0.059	0.031
Sprint Distance (m)	−0.072	−0.027	0.092	0.034	0.144

Note. Values represent Pearson’s r coefficients. ΔRT% = percentage change in reaction time, with positive values indicating slower responses and poorer performance; ΔRCS% = percentage change in Rate Correct Score, with negative values indicating poorer performance; ΔVariation% = percentage change in response variability, with positive values indicating greater response inconsistency and poorer performance; ΔAccuracy% = percentage change in accuracy, with negative values indicating poorer performance. M-VAS [Post] = post-match mental fatigue visual analogue scale score. HSR = high-speed running. * *p* < 0.05, ** *p* < 0.01.

**Table 4:** One-way ANOVAs: effect of match outcome on cognitive performance change scores.

Variable	F(2, 98)	*p*	η^2^p [90% CI]	M Loss	M Draw	M Win	Post Hoc
ΔReaction Time (%)	3.65	0.030	0.069 [0.000, 0.174]	18.65	2.95	13.42	Loss > Draw *
ΔSpeed (%)	3.76	0.027	0.071 [0.000, 0.177]	−11.2	−0.1	−7.4	Loss < Draw *
ΔRCS (%)	4.94	0.009	0.092 [0.007, 0.204]	−20.4	−4.6	−13.8	Loss < Draw **
ΔVariation (%)	0.89	0.414	0.018 [0.000, 0.086]	52.4	24.7	53.9	ns
ΔAccuracy (%)	0.52	0.597	0.010 [0.000, 0.067]	−0.9	−3.5	−4.3	ns

Note. η^2^p 90% CIs are reported (appropriate for F-tests). M Loss/Draw/Win = group mean change scores. Bonferroni correction applied across 3 pairwise contrasts. * *p* < 0.05, ** *p* < 0.01. ns = non-significant.

**Table 5:** Multiple regression: predictors of ΔVariation (%) from GPS external load metrics (*n* = 101).

Predictor	B	*β*	t	*p*	Tol.	VIF
Distance per minute (m·min^−1^)	0.870	0.128	1.193	0.236	0.813	1.23
HSR + Sprint Distance (m)	−0.040	−0.089	−0.832	0.407	0.820	1.22
High-intensity accelerations (>3 m·s^−2^)	1.212 **	0.468	2.911	0.004	0.360	2.78
High-intensity decelerations (<−3 m·s^−2^)	−0.500	−0.243	−1.465	0.146	0.338	2.96

Note. Exploratory observation-level multiple regression model predicting percentage change in response variation. Model: *R*^2^ = 0.108, adjusted *R*^2^ = 0.070, F(4, 96) = 2.90, *p* = 0.026. B = unstandardised coefficient; *β* = standardised coefficient; Tol. = tolerance. HSR = high-speed running. All VIFs < 3.0 indicate acceptable multicollinearity. Because repeated player–match measurements were nested within players, this model was re-examined using linear mixed-effects modelling in Section 3.8. ** *p* < 0.01.

**Table 6:** Linear mixed-effects models: fixed-effects omnibus tests for time, match outcome, and playing position on psychomotor and subjective fatigue outcomes (*n* = 202 measurements from 40 players).

Outcome	Time F (df)	*p*	Match Outcome F (df)	*p*	Playing Position F (df)	*p*	Marginal *R*^2^	Conditional *R*^2^	ICC
Reaction Time (ms)	49.75 (1, 154.5)	0.001	0.80 (2, 108.9)	0.451	1.22 (4, 32.8)	0.321	0.16	0.67	0.60
Variation (%)	36.44 (1, 161.1)	0.001	1.57 (2, 145.1)	0.211	0.32 (4, 35.9)	0.863	0.15	0.39	0.28
Accuracy (%)	6.18 (1, 156.7)	0.014	4.62 (2, 140.1)	0.011	0.90 (4, 28.3)	0.475	0.11	0.28	0.19
Speed	44.90 (1, 155.9)	0.001	2.57 (2, 106.6)	0.081	1.21 (4, 34.2)	0.326	0.18	0.70	0.64
Rate Correct Score	95.35 (1, 155.0)	0.001	1.02 (2, 120.6)	0.362	1.23 (4, 32.8)	0.316	0.25	0.63	0.51
Mental Fatigue VAS	2079.74 (1, 157.3)	0.001	0.03 (2, 138.0)	0.971	1.27 (4, 32.8)	0.302	0.88	0.92	0.33

Note. All models included time (pre, post), match outcome (loss, draw, win), playing position (CD, CM, F, SD, SM), and four GPS-derived load metrics (high-intensity accelerations (>3 m·s^−2^), high-intensity decelerations (<−3 m·s^−2^), distance·min^−1^, HSR + Sprint distance) as fixed effects, with a random intercept for player. Degrees of freedom were estimated using Satterthwaite’s approximation. ICC = intraclass correlation coefficient (between-player variance/total variance from null model). Marginal *R*^2^ reflects variance explained by fixed effects only; conditional *R*^2^ reflects variance explained by fixed and random effects combined.

## Data Availability

The data presented in this study are available upon reasonable request from the corresponding author because of restrictions.
